# *trans*-Dichloridobis(4-phenyl­pyridine-κ*N*)palladium(II)

**DOI:** 10.1107/S160053681105063X

**Published:** 2011-11-30

**Authors:** Kwang Ha

**Affiliations:** a School of Applied Chemical Engineering, The Research Institute of Catalysis, Chonnam National University, Gwangju 500-757, Republic of Korea

## Abstract

The asymmetric unit of the title complex, [PdCl_2_(C_11_H_9_N)_2_], contains one half of a neutral Pd^II^ complex, with the complete mol­ecule generated by the application of a twofold rotation axis; the N—Pd—N atoms lie on the axis. The Pd^II^ ion has a *trans*-Cl_2_N_2_ square-planar coordination geometry defined by two N atoms from two 4-phenyl­pyridine ligands and two Cl^−^ anions. In the refinement, the pyridine ring and the phenyl ring were found to be disordered over two sites with the site-occupancy factors being 0.53 (2) and 0.51 (1), respectively, for the major components.

## Related literature

For the crystal structure of the related Pt^II^ complex [PtCl_2_(C_11_H_9_N)_2_]·H_2_O, see: Ha (2011 ▶).
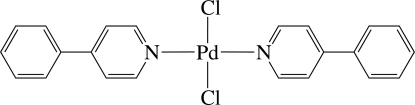


## Experimental

### 

#### Crystal data

[PdCl_2_(C_11_H_9_N)_2_]
*M* *_r_* = 487.68
Monoclinic, 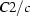

*a* = 9.4270 (15) Å
*b* = 23.680 (4) Å
*c* = 8.8554 (14) Å
β = 101.572 (3)°
*V* = 1936.6 (5) Å^3^
*Z* = 4
Mo *K*α radiation
μ = 1.24 mm^−1^
*T* = 200 K
0.21 × 0.08 × 0.06 mm

#### Data collection

Bruker SMART 1000 CCD diffractometer
Absorption correction: multi-scan (*SADABS*; Bruker, 2000 ▶) *T*_min_ = 0.875, *T*_max_ = 1.000
6848 measured reflections
2358 independent reflections
1866 reflections with *I* > 2σ(*I*)
*R*_int_ = 0.028

#### Refinement

*R*[*F* ^2^ > 2σ(*F* ^2^)] = 0.029
*wR*(*F* ^2^) = 0.077
*S* = 1.05
2358 reflections
165 parameters
H-atom parameters constrained
Δρ_max_ = 1.25 e Å^−3^
Δρ_min_ = −0.51 e Å^−3^

### 

Data collection: *SMART* (Bruker, 2000 ▶); cell refinement: *SAINT* (Bruker, 2000 ▶); data reduction: *SAINT*; program(s) used to solve structure: *SHELXS97* (Sheldrick, 2008 ▶); program(s) used to refine structure: *SHELXL97* (Sheldrick, 2008 ▶); molecular graphics: *ORTEP-3* (Farrugia, 1997 ▶) and *PLATON* (Spek, 2009 ▶); software used to prepare material for publication: *SHELXL97*.

## Supplementary Material

Crystal structure: contains datablock(s) global, I. DOI: 10.1107/S160053681105063X/tk5025sup1.cif

Structure factors: contains datablock(s) I. DOI: 10.1107/S160053681105063X/tk5025Isup2.hkl

Additional supplementary materials:  crystallographic information; 3D view; checkCIF report

## Figures and Tables

**Table 1:** Selected bond lengths (Å)

Pd1—N2	2.009 (3)
Pd1—N1	2.022 (3)
Pd1—Cl1	2.3018 (8)

**Fig. 1.**
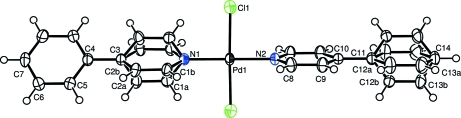
The disordered structure of the title complex, with displacement ellipsoids drawn at the 50% probability level. Unlabelled atoms are related to the reference atoms by the (-x, y, 3/2 - z) symmetry transformation.

**Fig. 2.**
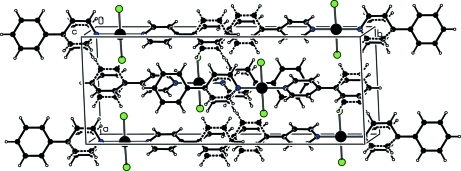
View of the unit-cell contents of the title complex. The minor bonds are drawn with dashed lines.

## supplementary crystallographic information

### Comment

The asymmetric unit of the title complex, [PdCl_2_(C_11_H_9_N)_2_], contains one half of a neutral Pd^II^ complex (Fig. 1). The complex is disposed about a twofold rotation axis running in the [010] direction passing through the Pd1, N1, C3, C4, C7, N2, C10, C11 and C14 atoms. The structure is similar to that of related Pt^II^ complex with a crystallization water, [PtCl_2_(C_11_H_9_N)_2_].H_2_O (Ha, 2011).

In the complex, the central Pd^II^ ion has a *trans*-Cl_2_N_2_ square-planar coordination geometry defined by two N atoms from two distinct 4-phenylpyridine ligands and two Cl^-^ anions. The two Pd—N bond lengths are nearly equal and the N—Pd—Cl bonds are almost perpendicular (Table 1). In the refinement, one pyridine ring (N1—C3) and one benzene ring (C11—C14) were found to be disordered over two sites. The dihedral angles between the major and minor rings are 33.2 (12)° for the ring N1—C3 and 42.2 (6)° for the ring C11—C14. The molecules stack in columns along the *a* axis and display numerous intermolecular π-π interactions between the six-membered rings, with a shortest ring centroid-centroid distance of 4.511 (6) Å (Fig. 2).

### Experimental

To a solution of Na_2_PdCl_4_ (0.2942 g, 1.000 mmol) in H_2_O (20 ml) and EtOH (10 ml) was added 4-phenylpyridine (0.3111 g, 2.005 mmol), followed by stirring for 3 h at room temperature. The formed precipitate was separated by filtration, washed with H_2_O and EtOH, and dried at 50 °C, to give a pale-yellow powder (0.4650 g). Crystals suitable for X-ray analysis were obtained by slow evaporation from its CH_3_CN solution.

### Refinement

H atoms were positioned geometrically and allowed to ride on their respective parent atoms [C—H = 0.95 Å and *U*_iso_(H) = 1.2*U*_eq_(C)]. One pyridyl ring (N1—C3) and one phenyl ring (C11—C14) displayed relatively large displacement factors so that the rings appear to be partially disordered. The C1, C2, C12 and C13 atoms were refined anisotropically as disordered over two sites, with the site-occupancy factors of 0.53 (2) and 0.51 (1) for the major components, respectively. The highest peak (1.25 e Å^-3^) and the deepest hole (-0.51 e Å^-3^) in the difference Fourier map are located 1.68 Å and 0.57 Å from the atoms H14 and Cl1, respectively.

### Crystal data

**Table:**

[PdCl_2_(C_11_H_9_N)_2_]	*F*(000) = 976
*M**_r_* = 487.68	*D*_x_ = 1.673 Mg m^−^^3^
Monoclinic, *C*2/*c*	Mo *K*α radiation, λ = 0.71073 Å
Hall symbol: -C 2yc	Cell parameters from 4007 reflections
*a* = 9.4270 (15) Å	θ = 2.4–28.3°
*b* = 23.680 (4) Å	µ = 1.24 mm^−^^1^
*c* = 8.8554 (14) Å	*T* = 200 K
β = 101.572 (3)°	Rod, yellow
*V* = 1936.6 (5) Å^3^	0.21 × 0.08 × 0.06 mm
*Z* = 4	

### Data collection

**Table:**

Bruker SMART 1000 CCD diffractometer	2358 independent reflections
Radiation source: fine-focus sealed tube	1866 reflections with *I* > 2σ(*I*)
graphite	*R*_int_ = 0.028
φ and ω scans	θ_max_ = 28.3°, θ_min_ = 1.7°
Absorption correction: multi-scan (*SADABS*; Bruker, 2000)	*h* = −12→11
*T*_min_ = 0.875, *T*_max_ = 1.000	*k* = −31→27
6848 measured reflections	*l* = −11→11

### Refinement

**Table:**

Refinement on *F*^2^	Primary atom site location: structure-invariant direct methods
Least-squares matrix: full	Secondary atom site location: difference Fourier map
*R*[*F*^2^ > 2σ(*F*^2^)] = 0.029	Hydrogen site location: inferred from neighbouring sites
*wR*(*F*^2^) = 0.077	H-atom parameters constrained
*S* = 1.05	*w* = 1/[σ^2^(*F*_o_^2^) + (0.038*P*)^2^] where *P* = (*F*_o_^2^ + 2*F*_c_^2^)/3
2358 reflections	(Δ/σ)_max_ < 0.001
165 parameters	Δρ_max_ = 1.25 e Å^−^^3^
0 restraints	Δρ_min_ = −0.51 e Å^−^^3^

### Special details

**Table:**

Geometry. All e.s.d.'s (except the e.s.d. in the dihedral angle between two l.s. planes) are estimated using the full covariance matrix. The cell e.s.d.'s are taken into account individually in the estimation of e.s.d.'s in distances, angles and torsion angles; correlations between e.s.d.'s in cell parameters are only used when they are defined by crystal symmetry. An approximate (isotropic) treatment of cell e.s.d.'s is used for estimating e.s.d.'s involving l.s. planes.
Refinement. Refinement of *F*^2^ against ALL reflections. The weighted *R*-factor *wR* and goodness of fit *S* are based on *F*^2^, conventional *R*-factors *R* are based on *F*, with *F* set to zero for negative *F*^2^. The threshold expression of *F*^2^ > σ(*F*^2^) is used only for calculating *R*-factors(gt) *etc*. and is not relevant to the choice of reflections for refinement. *R*-factors based on *F*^2^ are statistically about twice as large as those based on *F*, and *R*- factors based on ALL data will be even larger.

### Fractional atomic coordinates and isotropic or equivalent isotropic displacement parameters (Å^2^)

**Table:**

	*x*	*y*	*z*	*U*_iso_*/*U*_eq_	Occ. (<1)
Pd1	0.0000	0.125997 (10)	0.7500	0.02513 (11)	
Cl1	−0.23973 (8)	0.12738 (3)	0.76980 (10)	0.04242 (19)	
N1	0.0000	0.04062 (12)	0.7500	0.0249 (6)	
N2	0.0000	0.21085 (11)	0.7500	0.0251 (6)	
C1A	0.1164 (12)	0.0107 (3)	0.8189 (15)	0.034 (2)	0.53 (2)
H1A	0.2009	0.0306	0.8673	0.040*	0.53 (2)
C2A	0.1188 (12)	−0.0469 (3)	0.8225 (15)	0.033 (2)	0.53 (2)
H2A	0.2030	−0.0659	0.8754	0.040*	0.53 (2)
C1B	0.0744 (13)	0.0112 (3)	0.8716 (14)	0.028 (2)	0.47 (2)
H1B	0.1266	0.0316	0.9575	0.034*	0.47 (2)
C2B	0.0776 (12)	−0.0470 (4)	0.8759 (14)	0.030 (2)	0.47 (2)
H2B	0.1318	−0.0661	0.9631	0.036*	0.47 (2)
C3	0.0000	−0.07819 (14)	0.7500	0.0258 (8)	
C4	0.0000	−0.14083 (14)	0.7500	0.0274 (8)	
C5	0.1253 (3)	−0.17118 (10)	0.8113 (3)	0.0323 (6)	
H5	0.2114	−0.1514	0.8553	0.039*	
C6	0.1257 (3)	−0.22957 (11)	0.8090 (3)	0.0337 (6)	
H6	0.2127	−0.2496	0.8481	0.040*	
C7	0.0000	−0.25900 (15)	0.7500	0.0315 (9)	
H7	0.0000	−0.2991	0.7500	0.038*	
C8	0.0559 (3)	0.23996 (10)	0.8782 (3)	0.0312 (6)	
H8	0.0957	0.2198	0.9695	0.037*	
C9	0.0575 (3)	0.29803 (11)	0.8819 (3)	0.0316 (6)	
H9	0.0981	0.3171	0.9749	0.038*	
C10	0.0000	0.32924 (14)	0.7500	0.0242 (7)	
C11	0.0000	0.39200 (14)	0.7500	0.0260 (8)	
C12A	0.0602 (10)	0.4220 (2)	0.8820 (9)	0.038 (2)	0.512 (12)
H12A	0.1017	0.4022	0.9735	0.046*	0.512 (12)
C13A	0.0605 (9)	0.4803 (3)	0.8817 (10)	0.044 (2)	0.512 (12)
H13A	0.1025	0.5004	0.9727	0.053*	0.512 (12)
C12B	0.1230 (8)	0.4222 (2)	0.8248 (8)	0.0280 (17)	0.488 (12)
H12B	0.2060	0.4025	0.8778	0.034*	0.488 (12)
C13B	0.1226 (9)	0.4806 (2)	0.8206 (8)	0.0347 (19)	0.488 (12)
H13B	0.2072	0.5009	0.8666	0.042*	0.488 (12)
C14	0.0000	0.50968 (15)	0.7500	0.0344 (9)	
H14	0.0000	0.5498	0.7500	0.041*	

### Atomic displacement parameters (Å^2^)

**Table:**

	*U*^11^	*U*^22^	*U*^33^	*U*^12^	*U*^13^	*U*^23^
Pd1	0.02937 (17)	0.01596 (15)	0.02927 (17)	0.000	0.00399 (11)	0.000
Cl1	0.0372 (4)	0.0289 (4)	0.0612 (5)	0.0012 (3)	0.0102 (4)	−0.0014 (3)
N1	0.0261 (17)	0.0203 (15)	0.0278 (16)	0.000	0.0040 (13)	0.000
N2	0.0291 (17)	0.0183 (14)	0.0277 (16)	0.000	0.0049 (13)	0.000
C1A	0.026 (4)	0.027 (3)	0.044 (6)	0.000 (3)	−0.002 (3)	0.000 (3)
C2A	0.032 (4)	0.022 (3)	0.039 (5)	−0.002 (3)	−0.007 (4)	0.006 (3)
C1B	0.033 (5)	0.020 (3)	0.032 (4)	−0.004 (3)	0.006 (3)	−0.004 (3)
C2B	0.039 (5)	0.023 (3)	0.028 (4)	−0.003 (3)	0.008 (4)	0.005 (3)
C3	0.028 (2)	0.0191 (17)	0.032 (2)	0.000	0.0102 (16)	0.000
C4	0.033 (2)	0.0206 (17)	0.0303 (19)	0.000	0.0097 (16)	0.000
C5	0.0304 (16)	0.0244 (13)	0.0419 (17)	−0.0017 (10)	0.0065 (13)	−0.0008 (12)
C6	0.0352 (16)	0.0263 (14)	0.0404 (17)	0.0044 (11)	0.0093 (13)	0.0025 (12)
C7	0.045 (2)	0.0182 (18)	0.032 (2)	0.000	0.0114 (18)	0.000
C8	0.0406 (16)	0.0237 (13)	0.0268 (14)	0.0023 (11)	0.0005 (12)	0.0016 (10)
C9	0.0419 (17)	0.0247 (13)	0.0263 (14)	−0.0015 (11)	0.0021 (12)	−0.0027 (11)
C10	0.0251 (19)	0.0203 (17)	0.0280 (19)	0.000	0.0072 (15)	0.000
C11	0.033 (2)	0.0193 (17)	0.0285 (19)	0.000	0.0121 (16)	0.000
C12A	0.052 (5)	0.024 (3)	0.035 (4)	0.002 (3)	0.001 (3)	0.002 (2)
C13A	0.052 (5)	0.031 (3)	0.048 (4)	−0.003 (3)	0.006 (4)	−0.013 (3)
C12B	0.028 (4)	0.023 (3)	0.032 (4)	−0.001 (2)	0.004 (3)	0.000 (2)
C13B	0.046 (4)	0.023 (3)	0.037 (4)	−0.006 (3)	0.012 (3)	−0.008 (3)
C14	0.045 (3)	0.0193 (18)	0.041 (2)	0.000	0.013 (2)	0.000

### Geometric parameters (Å, °)

**Table:**

Pd1—N2	2.009 (3)	C6—C7	1.384 (3)
Pd1—N1	2.022 (3)	C6—H6	0.9500
Pd1—Cl1^i^	2.3018 (8)	C7—C6^i^	1.384 (3)
Pd1—Cl1	2.3018 (8)	C7—H7	0.9500
N1—C1A^i^	1.345 (8)	C8—C9	1.376 (3)
N1—C1A	1.345 (8)	C8—H8	0.9500
N1—C1B^i^	1.354 (9)	C9—C10	1.396 (3)
N1—C1B	1.354 (9)	C9—H9	0.9500
N2—C8	1.342 (3)	C10—C9^i^	1.396 (3)
N2—C8^i^	1.342 (3)	C10—C11	1.486 (5)
C1A—C2A	1.364 (10)	C11—C12A	1.389 (6)
C1A—H1A	0.9500	C11—C12A^i^	1.389 (6)
C2A—C3	1.388 (8)	C11—C12B	1.409 (6)
C2A—H2A	0.9500	C11—C12B^i^	1.409 (6)
C1B—C2B	1.381 (11)	C12A—C13A	1.381 (8)
C1B—H1B	0.9500	C12A—H12A	0.9500
C2B—C3	1.413 (9)	C13A—C14	1.379 (8)
C2B—H2B	0.9500	C13A—H13A	0.9500
C3—C2A^i^	1.388 (8)	C12B—C13B	1.384 (8)
C3—C2B^i^	1.413 (9)	C12B—H12B	0.9500
C3—C4	1.483 (5)	C13B—C14	1.383 (7)
C4—C5^i^	1.396 (3)	C13B—H13B	0.9500
C4—C5	1.396 (3)	C14—C13A^i^	1.379 (8)
C5—C6	1.383 (3)	C14—C13B^i^	1.383 (7)
C5—H5	0.9500	C14—H14	0.9500
			
N2—Pd1—N1	180.0	C5—C6—C7	120.3 (3)
N2—Pd1—Cl1^i^	89.182 (17)	C5—C6—H6	119.9
N1—Pd1—Cl1^i^	90.818 (17)	C7—C6—H6	119.9
N2—Pd1—Cl1	89.182 (17)	C6—C7—C6^i^	119.5 (3)
N1—Pd1—Cl1	90.818 (17)	C6—C7—H7	120.2
Cl1^i^—Pd1—Cl1	178.36 (3)	C6^i^—C7—H7	120.2
C1A^i^—N1—C1A	116.5 (7)	N2—C8—C9	122.3 (2)
C1A—N1—C1B^i^	109.6 (3)	N2—C8—H8	118.9
C1A^i^—N1—C1B	109.6 (3)	C9—C8—H8	118.9
C1B^i^—N1—C1B	118.1 (7)	C8—C9—C10	120.6 (2)
C1A^i^—N1—Pd1	121.8 (3)	C8—C9—H9	119.7
C1A—N1—Pd1	121.8 (3)	C10—C9—H9	119.7
C1B^i^—N1—Pd1	120.9 (4)	C9^i^—C10—C9	116.1 (3)
C1B—N1—Pd1	120.9 (4)	C9^i^—C10—C11	121.95 (16)
C8—N2—C8^i^	118.2 (3)	C9—C10—C11	121.95 (16)
C8—N2—Pd1	120.91 (15)	C12A—C11—C12A^i^	118.4 (6)
C8^i^—N2—Pd1	120.91 (15)	C12A^i^—C11—C12B	107.2 (4)
N1—C1A—C2A	123.0 (7)	C12A—C11—C12B^i^	107.2 (4)
N1—C1A—H1A	118.5	C12B—C11—C12B^i^	119.1 (5)
C2A—C1A—H1A	118.5	C12A—C11—C10	120.8 (3)
C1A—C2A—C3	121.0 (6)	C12A^i^—C11—C10	120.8 (3)
C1A—C2A—H2A	119.5	C12B—C11—C10	120.5 (3)
C3—C2A—H2A	119.5	C12B^i^—C11—C10	120.5 (3)
N1—C1B—C2B	122.7 (7)	C13A—C12A—C11	120.7 (6)
N1—C1B—H1B	118.6	C13A—C12A—H12A	119.6
C2B—C1B—H1B	118.6	C11—C12A—H12A	119.6
C1B—C2B—C3	119.7 (7)	C14—C13A—C12A	120.3 (6)
C1B—C2B—H2B	120.2	C14—C13A—H13A	119.8
C3—C2B—H2B	120.2	C12A—C13A—H13A	119.8
C2A—C3—C2A^i^	115.4 (7)	C13B—C12B—C11	119.8 (5)
C2A^i^—C3—C2B	109.2 (4)	C13B—C12B—H12B	120.1
C2A—C3—C2B^i^	109.2 (4)	C11—C12B—H12B	120.1
C2B—C3—C2B^i^	117.1 (8)	C14—C13B—C12B	120.5 (6)
C2A—C3—C4	122.3 (3)	C14—C13B—H13B	119.7
C2A^i^—C3—C4	122.3 (3)	C12B—C13B—H13B	119.7
C2B—C3—C4	121.5 (4)	C13A^i^—C14—C13A	119.5 (6)
C2B^i^—C3—C4	121.5 (4)	C13A—C14—C13B^i^	107.3 (4)
C5^i^—C4—C5	118.0 (3)	C13A^i^—C14—C13B	107.3 (4)
C5^i^—C4—C3	120.99 (16)	C13B^i^—C14—C13B	120.2 (6)
C5—C4—C3	120.99 (16)	C13A^i^—C14—H14	120.2
C6—C5—C4	120.9 (3)	C13A—C14—H14	120.2
C6—C5—H5	119.5	C13B^i^—C14—H14	119.9
C4—C5—H5	119.5	C13B—C14—H14	119.9
			
Cl1^i^—Pd1—N1—C1A^i^	−144.2 (8)	C2B—C3—C4—C5	−38.6 (7)
Cl1—Pd1—N1—C1A^i^	35.8 (8)	C2B^i^—C3—C4—C5	141.4 (7)
Cl1^i^—Pd1—N1—C1A	35.8 (8)	C5^i^—C4—C5—C6	1.06 (18)
Cl1—Pd1—N1—C1A	−144.2 (8)	C3—C4—C5—C6	−178.94 (18)
Cl1^i^—Pd1—N1—C1B^i^	−110.4 (7)	C4—C5—C6—C7	−2.1 (4)
Cl1—Pd1—N1—C1B^i^	69.6 (7)	C5—C6—C7—C6^i^	1.05 (18)
Cl1^i^—Pd1—N1—C1B	69.6 (7)	C8^i^—N2—C8—C9	−0.01 (19)
Cl1—Pd1—N1—C1B	−110.4 (7)	Pd1—N2—C8—C9	179.99 (19)
Cl1^i^—Pd1—N2—C8	−79.23 (14)	N2—C8—C9—C10	0.0 (4)
Cl1—Pd1—N2—C8	100.77 (14)	C8—C9—C10—C9^i^	−0.01 (18)
Cl1^i^—Pd1—N2—C8^i^	100.77 (14)	C8—C9—C10—C11	179.99 (18)
Cl1—Pd1—N2—C8^i^	−79.23 (14)	C9^i^—C10—C11—C12A	−178.8 (5)
C1A^i^—N1—C1A—C2A	−0.9 (5)	C9—C10—C11—C12A	1.2 (5)
C1B^i^—N1—C1A—C2A	−31.3 (6)	C9^i^—C10—C11—C12A^i^	1.2 (5)
C1B—N1—C1A—C2A	81.7 (13)	C9—C10—C11—C12A^i^	−178.8 (5)
Pd1—N1—C1A—C2A	179.1 (5)	C9^i^—C10—C11—C12B	−137.3 (4)
N1—C1A—C2A—C3	1.8 (9)	C9—C10—C11—C12B	42.7 (4)
C1A^i^—N1—C1B—C2B	30.4 (6)	C9^i^—C10—C11—C12B^i^	42.7 (4)
C1A—N1—C1B—C2B	−79.2 (13)	C9—C10—C11—C12B^i^	−137.3 (4)
C1B^i^—N1—C1B—C2B	0.2 (5)	C12A^i^—C11—C12A—C13A	−0.2 (5)
Pd1—N1—C1B—C2B	−179.8 (5)	C12B—C11—C12A—C13A	79.4 (8)
N1—C1B—C2B—C3	−0.5 (9)	C12B^i^—C11—C12A—C13A	−36.9 (6)
C1A—C2A—C3—C2A^i^	−0.9 (4)	C10—C11—C12A—C13A	179.8 (5)
C1A—C2A—C3—C2B	−83.7 (14)	C11—C12A—C13A—C14	0.4 (10)
C1A—C2A—C3—C2B^i^	28.3 (6)	C12A—C11—C12B—C13B	−80.1 (8)
C1A—C2A—C3—C4	179.1 (4)	C12A^i^—C11—C12B—C13B	35.0 (5)
C1B—C2B—C3—C2A	79.8 (13)	C12B^i^—C11—C12B—C13B	−1.5 (4)
C1B—C2B—C3—C2A^i^	−28.6 (6)	C10—C11—C12B—C13B	178.5 (4)
C1B—C2B—C3—C2B^i^	0.2 (4)	C11—C12B—C13B—C14	3.1 (8)
C1B—C2B—C3—C4	−179.8 (4)	C12A—C13A—C14—C13A^i^	−0.2 (5)
C2A—C3—C4—C5^i^	174.0 (8)	C12A—C13A—C14—C13B^i^	38.0 (6)
C2A^i^—C3—C4—C5^i^	−6.0 (8)	C12A—C13A—C14—C13B	−79.6 (8)
C2B—C3—C4—C5^i^	141.4 (7)	C12B—C13B—C14—C13A^i^	−39.6 (6)
C2B^i^—C3—C4—C5^i^	−38.6 (7)	C12B—C13B—C14—C13A	76.8 (8)
C2A—C3—C4—C5	−6.0 (8)	C12B—C13B—C14—C13B^i^	−1.6 (4)
C2A^i^—C3—C4—C5	174.0 (8)		

Symmetry codes: (i) −*x*, *y*, −*z*+3/2.
